# Effects of Electrode Material on the Voltage of a Tree-Based Energy Generator

**DOI:** 10.1371/journal.pone.0136639

**Published:** 2015-08-24

**Authors:** Zhibin Hao, Guozhu Wang, Wenbin Li, Junguo Zhang, Jiangming Kan

**Affiliations:** School of Technology, Beijing Forestry University, Beijing, China; Tsinghua University, CHINA

## Abstract

The voltage between a standing tree and its surrounding soil is regarded as an innovative renewable energy source. This source is expected to provide a new power generation system for the low-power electrical equipment used in forestry. However, the voltage is weak, which has caused great difficulty in application. Consequently, the development of a method to increase the voltage is a key issue that must be addressed in this area of applied research. As the front-end component for energy harvesting, a metal electrode has a material effect on the level and stability of the voltage obtained. This study aimed to preliminarily ascertain the rules and mechanisms that underlie the effects of electrode material on voltage. Electrodes of different materials were used to measure the tree-source voltage, and the data were employed in a comparative analysis. The results indicate that the conductivity of the metal electrode significantly affects the contact resistance of the electrode-soil and electrode-trunk contact surfaces, thereby influencing the voltage level. The metal reactivity of the electrode has no significant effect on the voltage. However, passivation of the electrode materials markedly reduces the voltage. Suitable electrode materials are demonstrated and recommended.

## Introduction

When two metal electrodes are installed in a tree and the adjacent soil, a voltage develops between the electrodes [1]. This voltage has been regarded as an innovative renewable energy generator. Compared with other natural energy sources, such as solar energy and wind energy, this type of energy has certain advantages, including eco-friendliness, a long lifetime, and no restrictions with respect to the hermetic environment in the forest. Hence, scholars have forecasted practical applications of the tree-voltage that include a wide variety of trickle chargers for niche environmental sensors and mesh-networked devices, drastically decreasing the need for in-the-field battery changes [2]. Sensors and networks used in forestry have extremely low energy consumption as a result of their low-power design and sleep function. The tree-based voltage has the ability to provide uninterruptible energy when charging a low-voltage battery to meet the demands of discontinuous power consumption. Therefore, the tree-based voltage is a promising energy source. This source is expected to provide a brand-new power generation system for the low-power electrical equipment used in forestry. However, studies have shown that this type of energy is weak, i.e., the voltages obtained are typically in the tens to hundreds of millivolts range, while the short circuit currents are in the microampere range [2]. A low electrical power in the microwatt range (approximately 20 μW) was measured in a previous study. These features are insufficient for directly driving the low-power electronic equipment currently used in forests.

The technology for harvesting and utilizing micro-energy, which includes piezoelectric energy, photovoltaic energy, and thermoelectric energy, is relatively well-developed [3–8]. To acquire the suitable voltage level necessary to charge a storage circuit and subsequently to power electronic devices, a boosting operation with a direct current-to-direct current (DC-DC) converter is generally required [9, 10]. However, the voltage between a standing tree and its surrounding soil is quite low and cannot reliably drive the present DC-DC circuit modules. That is, the existing challenge in this regard is to increase the voltage. To solve this problem, relevant studies have focused on two major topics: 1) ascertaining the mechanism behind this voltage [2], thereby determining whether the voltage may be artificially increased, and 2) finding novel boosting methods while designing innovative low-voltage electronics [11, 12].

Research on the mechanism of tree-based energy is an important task. It is well-known that electric potential differences exist in plant bodies. However, this type of voltage is typically caused by the physiological activities of plants [13–16]. This phenomenon can be explained at a microscopic level, in particular at the cellular level. The electric potential difference caused by physiological activities is extremely weak (at most tens of millivolts) and sometimes transient (generated in response to external stimuli) [17]. This is negligible in studies of the tree-soil voltage, which is typically in the tens to hundreds of millivolts range. Studies of the voltage that arises between a standing tree and its surrounding soil belong to a new field of research. Early observations of tree trunk electrical activity were confirmed by an experiment on a spruce tree [18], which further reported a non-linear relationship between the electric potential variation and the intensity of the solar radiation. Some scholars have claimed that sap flow is the dominating mechanism for voltage generation [19]. Moreover, other scholars have proposed a mechanism based on charge diffusion from the conductive sap flow channels into the resistive xylem walls [18]. However, electrical signals with more erratic time variations were still observed in the experiments, indicating that other mechanisms, possibly unrelated to sap flow, may also contribute. Atmospheric electricity was claimed to contribute to temporal voltage variations. However, inherent voltages were also observed in those studies [20–22]. In addition, it was found that the pH difference between the interior of the tree and its surrounding soil significantly affects the voltage [2]. Therefore, the mechanism behind the tree-based energy generator remains controversial, leading to significant unknown factors that continue to affect its application.

The invention of innovative electronics is another objective that must be met to utilize tree-based energy. A DC-DC converter can boost the input voltage and generate constant output, but such a circuit requires a minimum threshold voltage as the input, which a standing tree cannot reliably generate. In response to this problem, some researchers have designed a boost-circuit that has low power consumption and a low input voltage, as well as a low-power clock circuit. Both circuits have been driven by a standing tree with relatively good performance [11].

However, to utilize this type of electricity more reliably, various factors, such as the harvesting, conversion, and storage of electricity, must be considered. During the process of utilizing standing-tree electricity, electrodes should be arranged in the standing tree and in the surrounding soil, as the front-end equipment for energy harvesting. However, the electrode material may affect the magnitude and stability of the electricity obtained. Because the mechanism behind the tree-based voltage remains unclear, no universal standard has been established for electrode selection during the process of measuring and utilizing tree electricity [2, 11, 12, 18–22]. However, metal electrodes remain the preferred material of scientists, based on the relevant literature. Most previous studies have used steel electrodes [11, 12, 18–22]. Moreover, platinum (Pt) electrodes have also been used in some studies [2]. Regardless, to take advantage of such a weak energy source with high efficiency, the choice of a suitable electrode is a central issue.

The stability (metal reactivity) of the electrode material is an important factor for consideration because the trunk and soil can cause electrode corrosion. Steel and Pt electrodes offer good corrosion resistance, which yields a stable electrical signal when measured over time. However, it has not yet been shown whether the stability of the materials significantly affects the voltage during collection and utilization. Moreover, the process requires the highest possible voltage during power collection and utilization. Therefore, conductivity is also an important factor. In a previous study, a higher voltage was measured using electrodes with a lower resistivity, i.e., better conductivity [23]. However, the power source formed by a standing tree and its surrounding soil has a high internal resistance. Internal resistances of tens of thousands of ohms have been observed, according to the relevant literature [11, 12, 24]. The metal electrodes act as a series resistor with a low resistance between the anode and cathode of the tree-source. In this case, the conductivity of the electrode material exerts little effect on the output voltage, which is inconsistent with the previous study. Therefore, the mechanism by which the electrode material affects the voltage requires further study. This work aimed to preliminarily ascertain the rules and mechanisms that underlie the effect of the electrode material on the voltage between a standing tree and its surrounding soil, as well as to provide guidance for choosing suitable electrode materials in relevant studies, thereby promoting the utilization of this innovative energy generator. Both the conductivity and the stability of the metal electrodes studied here were considered.

## Materials and Methods

### Experimental electrodes

Based on the objective of this study, three different materials with different conductivities and stabilities, including copper (Cu), aluminum (Al), and iron (Fe), were selected. The conductivities of Cu, Al, and Fe are different, varying in the order of Cu to Al and Fe for strong to weak conductivity. The resistivities of the three materials are approximately 1.75 × 10^-8 Ωm (Cu), 2.83 × 10^-8 Ωm (Al), and 9.78 × 10^-8 Ωm (Fe). The reactivities are also different, varying in the order of Cu to Fe and Al for low to high reactivity. Significantly different values for both resistivity and metal reactivity exist among the three metals. All electrodes were of the same size, with a diameter of 0.6 cm and a length of 8 cm. For the convenience of comparison, two additional electrodes (Al and Fe) of the same size were chosen. However, these two electrodes were treated with a 10-μm-thick Cu plating, which caused a negligible effect on the electrode size. Moreover, artificial damage (a minor scratch) was applied to the Cu plating of the Cu-plated Al electrode, which was employed as a control in the experiment. The purpose in this case was to ascertain the effect of the Cu plating.

### Electrode arrangement

**Fig 1:**
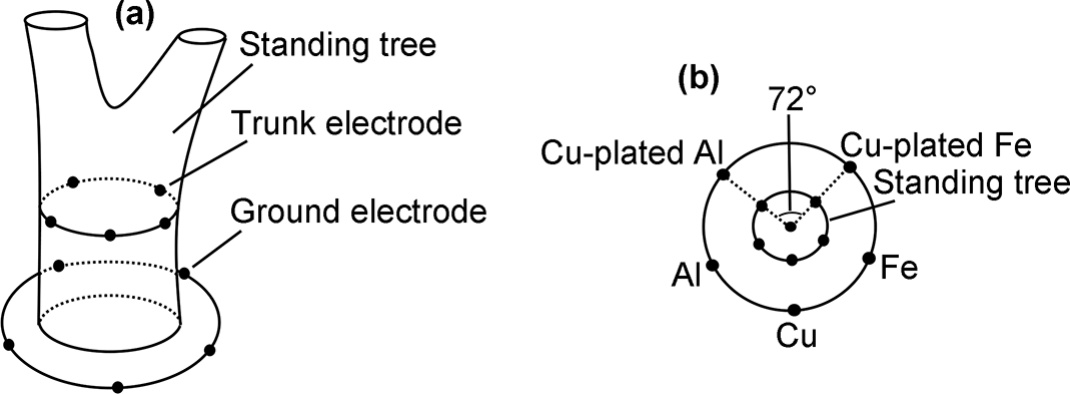
Electrode arrangement. (a) Five different electrodes were inserted into the trunk. Another five electrodes were arranged in the soil surrounding the standing tree. (b) In both sets, the electrodes were in the order of Cu-plated Al, Al, Cu, Fe, and Cu-plated Fe. Lines connecting any two adjacent electrodes to the center of the trunk had an angle of 72°.

A naturally grown, healthy poplar (*Populus X canadensis Moench*) with a diameter of 46 cm (at the height of 0.5 m where the electrodes were arranged) was chosen, and five electrodes (trunk electrodes) were inserted into its trunk at even spacing in the order of Cu-plated Al, Al, Cu, Fe, and Cu-plated Fe (the electrode position has no effect on the voltage [2]), as shown in Fig 1. The internal end of the electrode was implanted to a depth of up to 5 cm into the trunk, and the external end was attached to a wire. Another five electrodes (ground electrodes) were evenly arranged in the soil surrounding the standing tree in the same order. The ground electrodes were inserted to a depth of 80 cm [18]. Lines connecting any two adjacent electrodes to the center of the trunk had an angle of 72°. The ground surrounding the tree was flat, and the nearby soil composition was uniform.

### Measurements

A wire was introduced from each trunk electrode and its corresponding ground electrode. A load resistor was connected to the wires between the trunk electrode and the ground electrode in series so that a current circuit was formed through the load resistor [11]. The voltage across the resistor was measured using a high-input impedance voltmeter (Keithley 2000).

It has been previously observed that the electrical activity of trees strongly depends on the season, with more stable and coherent voltage variations measured during spring and summer [18]. Therefore, the tree-based energy generator is expected to operate during spring and summer. The present study was conducted during summer (June-August 2014). Every measurement was conducted at noon.

The experimental site located in an experimental forest in Beijing Forestry University for which specific permission was not required. The field studies did not involve endangered or protected species.

### The electrode-soil contact resistance

**Fig 2:**
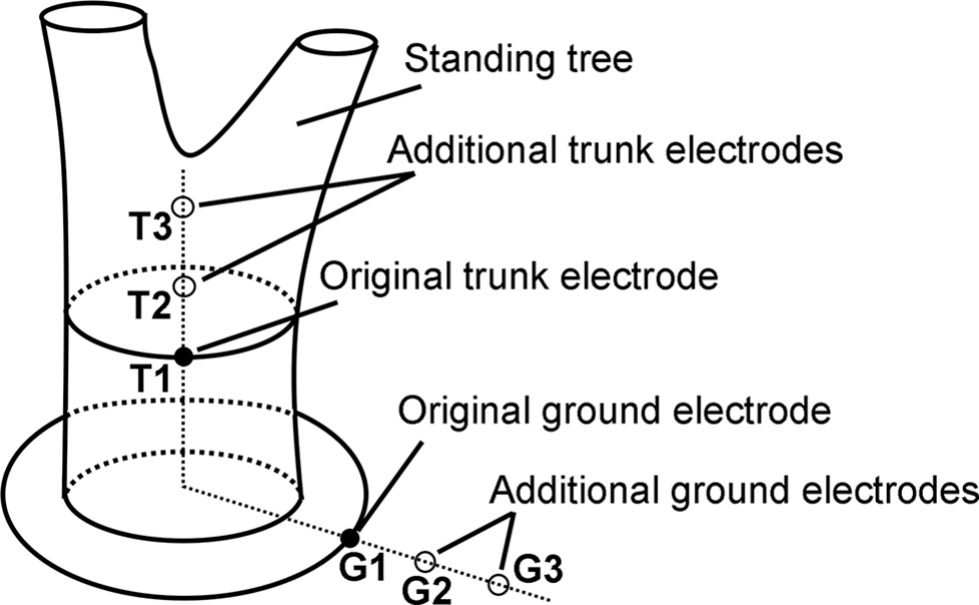
The first additional experiment. T1 and G1 denote the original Cu electrode in the trunk and the original Cu electrode in the soil, respectively. T2 and T3 are the additional Cu electrodes in the trunk. G2 and G3 are the additional Cu electrodes in the soil.

To ascertain the effect of the electrode-soil resistance on the internal resistance of the power source formed by a standing tree and its surrounding soil, an additional experiment (the first additional experiment) was performed. This experiment was undertaken to help explain the effect of the electrode conductivity on the voltage. As shown in Fig 2, two additional Cu electrodes were arranged in the trunk with a separation of 30 cm, while two additional Cu electrodes were arranged in the soil with a separation of 30 cm.

T1 and G1 denote, respectively, the original Cu electrode in the standing tree and the original Cu electrode in the soil. T2 and T3 are the additional Cu electrodes in the trunk. G2 and G3 are the additional Cu electrodes in the soil. Each measurement of the voltage between the trunk electrodes and the ground electrodes, as captured using the load resistor, was recorded as V_T1-G1_, V_T1-G2_, V_T1-G3_, V_T2-G1_, V_T2-G2_, V_T2-G3_, V_T3-G1_, V_T3-G2_, and V_T3-G3_. After tamping the surface soil at G1, G2, and G3, the voltages were recorded again.

### The electrode-trunk contact resistance

**Fig 3:**
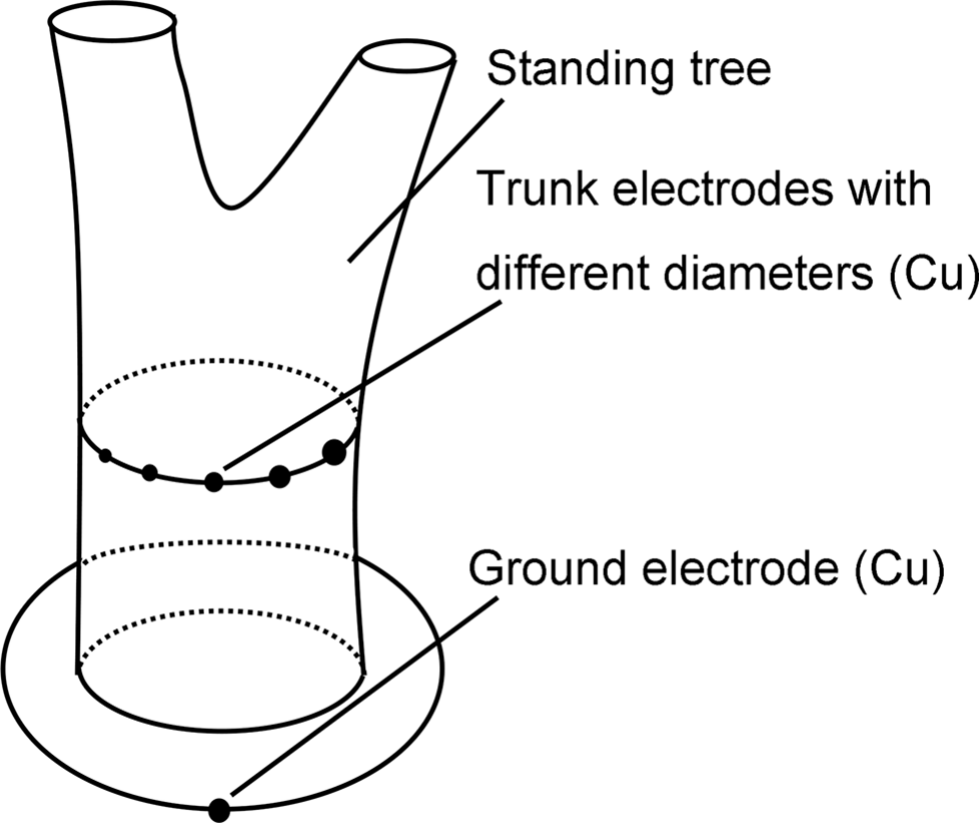
The second additional experiment. Five cylindrical Cu electrodes with different diameters were inserted into the tree trunk. Another Cu electrode was arranged in the soil.

To ascertain the effect of the contact resistance between the electrode and the trunk on the internal resistance of the tree-source, a second additional experiment was conducted. As shown in Fig 3, five cylindrical Cu electrodes (trunk electrodes) with different diameters (0.5 cm, 1.0 cm, 1.5 cm, 2.0 cm, and 2.5 cm) were inserted into the tree trunk. These electrodes were all implanted at the same depth (5 cm) in the trunk. As a result, the electrodes had different contact areas with the trunk. Another Cu electrode (ground electrode) was arranged in the soil. The voltages between each trunk electrode and the ground electrode were measured.

### Electrodes with nonmetallic inclusions

**Fig 4:**
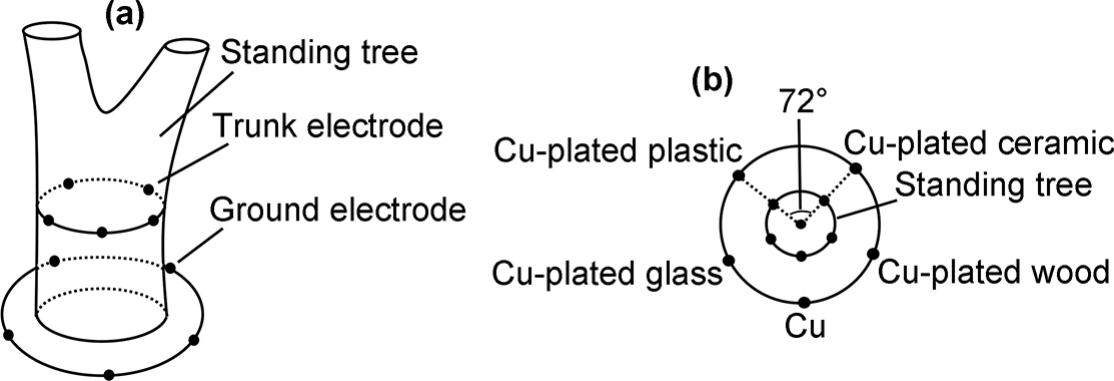
Supplementary experiment. (a) Five different electrodes were inserted into the trunk. Another five different electrodes were arranged in the soil surrounding the standing tree. (b) The electrodes were in the order of Cu-plated plastic, Cu-plated glass, Cu, Cu-plated wood, and Cu-plated ceramic.

A supplementary experiment was conducted to test the performance of electrodes with nonmetallic inclusions. This supplementary experiment was similar to the primary experiment. However, four nonmetallic materials were used as the inclusions in the supplementary experiment (Fig 4). Moreover, the thickness of the coating was 25 μm, which was thicker than that in the primary experiment.

## Results

### Primary experiment

**Fig 5:**
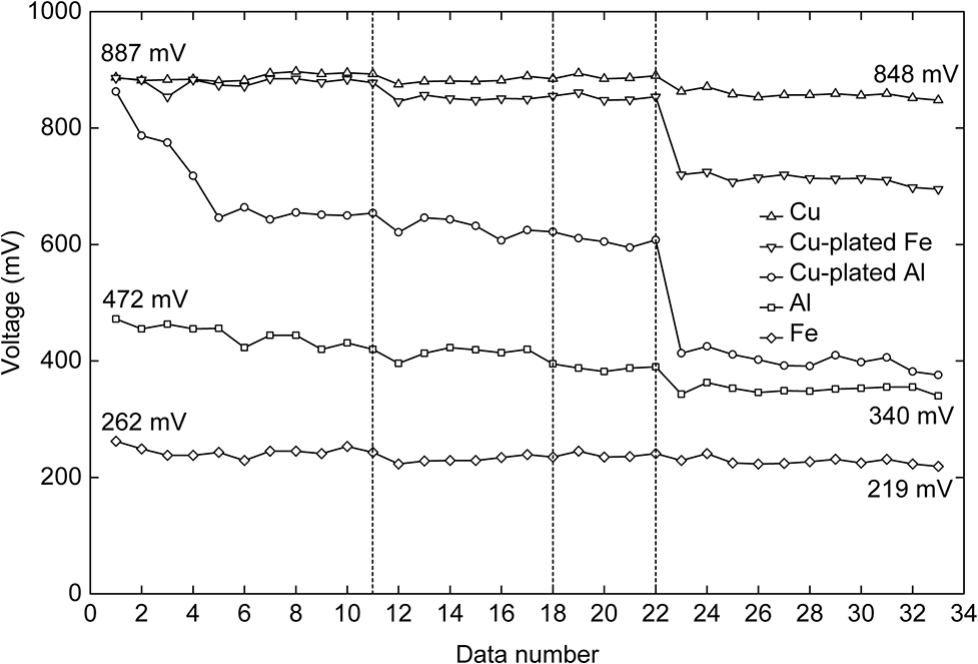
Voltage variations in the primary experiment. Five curves display the voltage variations measured with the five different electrodes. All curves show an overall decreasing trend. The Cu electrode yields the highest measured voltage, followed by Al and Fe. The curve acquired from the Cu-plated Fe electrode is closest to that of the Cu electrode. The curve of the Cu-plated Al electrode declines sharply.

Thirty-three sets of effective data were acquired from the primary experiment. The voltage variations are shown in Fig 5. The y-axis displays the voltages measured, and the x-axis shows the serial number of each data set, which varies with time. The voltage measured by the three metal electrodes (Cu, Al, and Fe) did not show any specificity except for differences in the magnitude and amplitude of variation. The Al electrode demonstrated a slightly different voltage trend relative to the other two metals. Among the three metals (Cu, Al, and Fe), the Cu electrode yielded the highest measured voltage, followed by Al and Fe. The curves for the three metal electrodes, i.e., Cu, Al and Fe, all fluctuated but showed an overall decreasing trend, and the final recorded point in each case was the lowest. For the Cu electrode, the last data point was 848 mV, representing a decrease of 39 mV from the first recorded point (887 mV). For the Al electrode, the voltage decreased by 132 mV. For the Fe electrode, the voltage decreased by 43 mV. The voltage measured by the Cu electrode decreased the least, followed by the Fe electrode, whereas the Al electrode registered the largest decrease in voltage. The voltage curve acquired from the Cu-plated Fe electrode was closest to that of the Cu electrode. These two curves were nearly identical, especially at the data sets prior to group 22. Likewise, the Cu-plated Al electrode also demonstrated properties similar to the Cu electrode at the first data point. However, its voltage curve then approached the curve of the Al electrode.

### The electrode-soil contact resistance

**Fig 6:**
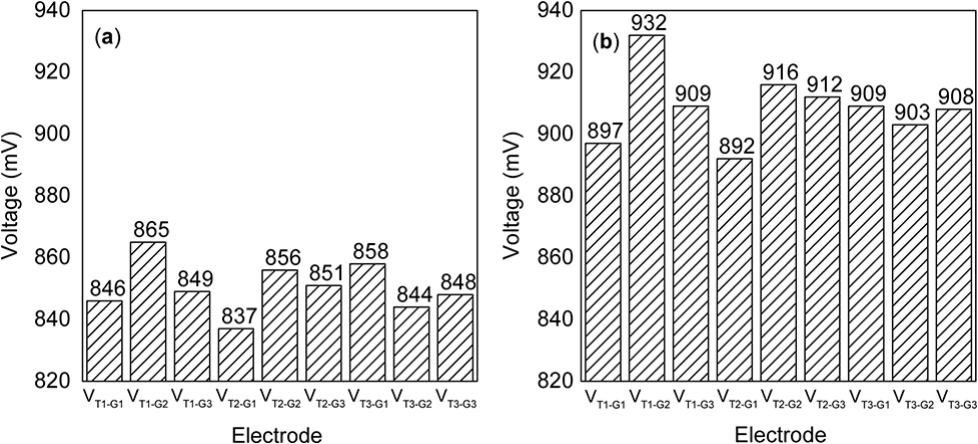
Voltages recorded in the first additional experiment. Each measurement of the voltage between the electrodes in the tree trunk and the surrounding soil using the load resistor was recorded as V_T1-G1_, V_T1-G2_, V_T1-G3_, V_T2-G1_, V_T2-G2_, V_T2-G3_, V_T3-G1_, V_T3-G2_, and V_T3-G3_. (a) The voltage had no correlation with the position shift of the electrodes. (b) All voltages increased after the soil was tamped.

The results from the first additional experiment are presented in Fig 6. Fig 6A shows the voltages before the soil was tamped. The voltages after tamping are presented in Fig 6B. There are no common differences among the voltages in Fig 6A. The voltage change did not correlate with the position shift of the electrodes. In Fig 6B, every voltage is greater than the voltages shown in Fig 6A.

### The electrode-trunk contact resistance

**Fig 7:**
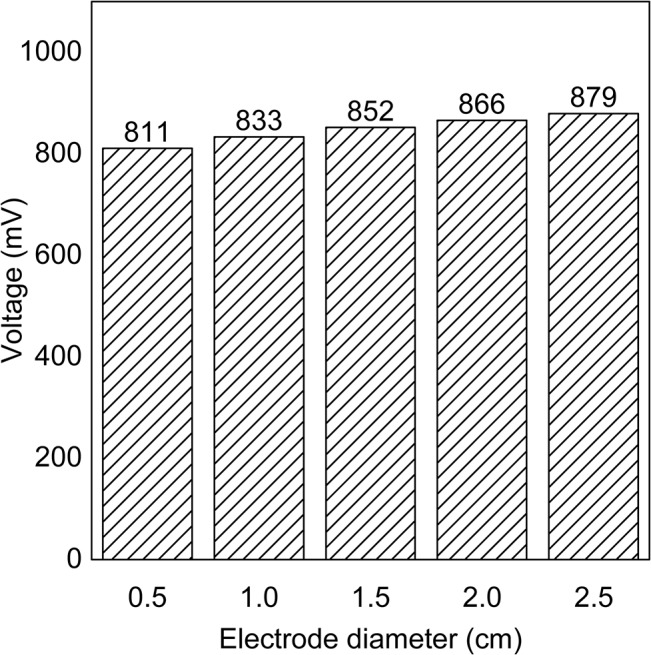
Voltages recorded in the second additional experiment. The electrode with the largest diameter obtained the highest voltage.

The result of the second additional experiment is presented in Fig 7. This figure shows the voltages measured by the trunk electrodes of different diameters, which varied from 0.5 cm to 2.5 cm in 0.5 cm intervals. The voltage rose discernably with increasing electrode diameter. That is, the measured voltage increased with electrode diameter (increasing contact area with the tree trunk).

### Electrodes with nonmetallic inclusions

**Fig 8:**
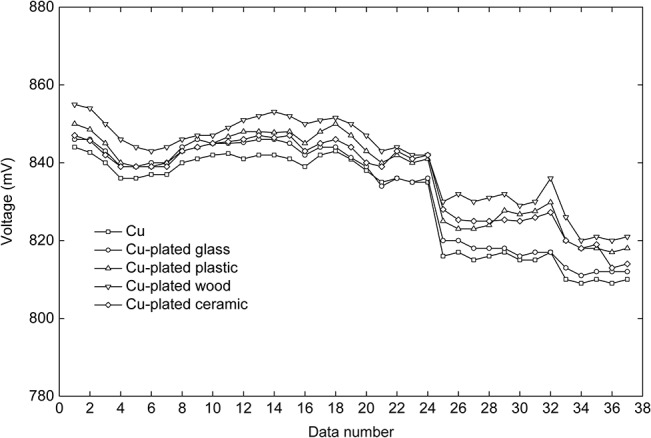
Voltage variations in the supplementary experiment. Five curves display the voltage variations measured with the five different electrodes. All curves follow the same trend. There is no obvious difference in amplitude among the curves.

The result of the supplementary experiment is shown in Fig 8. The y-axis displays the voltages and the x-axis shows the serial number of each data set, which varies with time. The curves in Fig 8 all follow the same trend. The voltage drops of the Cu-plated electrodes were smaller than those recorded in the primary experiment (Fig 5). There is no obvious difference in amplitude between the Cu electrode and the Cu-plated electrodes. However, the voltages measured by the Cu-plated electrodes were slightly higher than those of the Cu electrode.

## Discussion

### Conductivity

The magnitudes of the voltages measured with the three different electrodes (Cu, Al, and Fe) in the primary experiment, from large to small, were in the order of Cu to Al and Fe, which is consistent with the conductivities of the three metals (Fig 5). However, the internal resistance of the power source formed by the standing tree and the soil is large (at least 17.9 kΩ in the present study). The electrodes act as a part of the source. However, because each electrode has a small resistance, it should not affect the size of the internal resistance and the measured voltage. Therefore, the difference in the resistance of the three electrodes was not the immediate cause of the different voltages. To uncover the actual phenomenon, two additional experiments were conducted (Figs 2 and 3).

In the first additional experiment (Fig 2), the distance between the electrodes was increased from position T1 to position T3 in the standing tree and from G1 to G3 in the soil (Fig 2). The corresponding data (Fig 6A) revealed that the voltage change did not correlate with the shift in position, indicating that the position of an electrode does not strongly affect the internal resistance of the tree-source. Therefore, the contributions from the standing tree and the soil to the internal resistance are negligible. However, when the surface soil at G1, G2, and G3 was tamped, all corresponding voltages increased by at least 50 mV (Fig 6B). Likewise, tamping the surface soil of the Al, Fe, Cu-plated Al, and Cu-plated Fe electrodes also produced a similar effect on the voltage. This finding suggests that the contact resistance between an electrode and the soil is a major component of the internal resistance of the tree-based power source. In the second additional experiment (Fig 3), higher voltages were obtained from electrodes with larger diameters (Fig 7). That is, increasing the contact area between the electrode and the trunk also increased the measured voltage. This finding suggests that the contact resistance between an electrode and tree trunk is also an important part of the internal resistance of the tree-source. The value of this contact resistance may be thousands of ohms, in consideration of the high level of internal resistance present in the tree-source (in the range of 15.9 kΩ to 21.1 kΩ in the second additional experiment). Therefore, the reason for the different voltages among the three electrodes (Cu, Al, and Fe, Fig 5) is the difference in the contact resistance of electrode-soil and electrode-trunk contact surfaces and not the difference in the electrode resistance alone. That is, smaller contact resistances between the electrode and the soil (or trunk) caused higher voltages. Moreover, under the same soil and trunk environment, the metal with the higher conductivity also achieved a smaller contact resistance with the soil and the trunk. Therefore, the Cu electrode (which has the highest conductivity) achieved a smaller contact resistance with the soil and the trunk than Al electrode and Fe electrode, resulting in the highest voltage obtained in the primary experiment (Fig 5). The internal resistance of the tree-source was also measured by all three metal electrodes (Cu, Al, and Fe). The measured resistances were 17.9 kΩ (Cu), 105.7 kΩ (Al), and 188.9 kΩ (Fe). The magnitudes of the resistances measured with the three different electrodes (Cu, Al, and Fe), from small to large, were Cu, Al, and Fe, which was consistent with the resistivities of the three metals. Moreover, the metal with the highest conductivity (Cu) decreased the internal resistance (and the contact resistance) by tens of thousands of ohms. This result indicated that the metal with the higher conductivity achieved a smaller contact resistance and smaller internal resistance of the tree-source. In this case, the effects of metal conductivity on voltage are primarily determined by the surface metal materials in direct contact with the soil and the trunk. The primary experiment also demonstrated that cheap metals, such as Fe and Al, can be selected for inclusion by coating a more conductive metal, such as Cu. As an extension of this concept, a supplementary experiment was conducted to test the performance of electrodes with nonmetallic inclusions (Fig 4).

In the supplementary experiment (Fig 4), the magnitudes of the voltages measured by the electrodes with nonmetallic inclusions (plastic, glass, wood, and ceramic) were similar to that of the Cu electrode (Fig 8). These results indicate that nonmetallic materials, such as plastic, glass, wood, and ceramic, can also be good choices for electrode construction. In addition, when Cu is plated on the surface of a nonmetallic material, the resulting surface is slightly rougher than when Cu is plated on a metal. This surface roughness can increase the contact area between the electrode and the soil. Therefore, higher voltages were measured by the Cu-plated electrodes in the supplementary experiment.

### Stability

The resistivity of Al is closer to that of Cu than Fe, but the voltage curve measured with the Al electrode was closer to that of the Fe electrode (Fig 5). This result was obtained because Al oxidizes more readily, forming a high-resistance oxide layer [25], which increases the contact resistance between the electrode and the soil (and the trunk). As a result, the voltage curve measured with the Al electrode was closer to that of the Fe electrode. In addition, the Al electrode exhibited significant instability throughout the entire experiment, also yielding the most rapidly decreasing voltage curve. In addition to oxidation, Al also undergoes a process of self-passivation, in which a compact oxide coating forms on its surface [26]. This phenomenon likely occurred in the experiment, reducing the contact area between the Al metal and the soil (and the trunk). In contrast, the metals Cu and Fe are resistant to passivation in natural or mildly acidic soil and tree trunk conditions, hence these electrodes resulted in relatively flat corresponding curves. Fe can oxidize rapidly in wet environments; however, in this experiment, the corresponding voltage decrease was slightly larger than that of Cu, but not as significant as that of Al. The reason for this is that no passivation occurred on the surface of the Fe electrode. That is, although a large oxidized area developed on the Fe surface, the oxide did not form a coherent oxide layer. Therefore, the contact area between the Fe electrode and the soil (and the trunk) or the moisture in the soil (and the trunk) did not significantly decrease. Furthermore, due to the specific nature of the Al electrode, as discussed, its corresponding voltage curve differed from those of the Fe and Cu electrodes. For example, data sets 17 to 19 (Fig 5) for the Cu and the Fe electrodes display slowly increasing voltages, whereas the corresponding curve for the Al electrode decreases continuously. These results suggest that during such a period, factors associated with either the environment or the trunk of the standing tree caused the voltage to increase, while the passivation of the Al electrode severely affected the voltage ultimately obtained. Thus, the stability of the electrode material has no significant effect on the voltage. However, passivation of the electrode material can significantly reduce the voltage obtained.

### Cu plating

Higher voltages were measured for the Cu-plated Al and Fe electrodes compared with the non-plated Al and Fe electrodes. When the first measurement was recorded (Fig 5), each electrode was in its initial state such that the difference in the measured voltage was determined by the conductivity of each electrode. The measured voltages recorded with Cu, Cu-plated Al, and Cu-plated Fe were almost identical, indicating that the Cu plating on the Al and Fe electrodes provided these complex electrodes with nearly the same characteristics as the Cu electrode. It is easy to comprehend that after plating with Cu, the Al and Fe electrodes acquired surface and contact resistances with the soil and trunk that were similar to those of the Cu electrode. However, as revealed by the first data set, the Cu-plated Al electrode had a smaller voltage because the plating was artificially damaged before the experiment. Because the degree of damage was limited, this electrode’s voltage was not markedly different from that of either the Cu electrode or the Cu-plated Fe electrode. However, after time had passed, the voltage curve of the Cu-plated Al electrode decreased significantly and eventually merged with the corresponding curve of the Al electrode. In contrast, the Cu-plated Fe electrode showed little decrease in voltage before data set 11. These results suggest that in these wet soil and trunk environments, the minor damage to the Cu coating of the Cu-plated Al electrode before the experiment resulted in the rapid degradation of the plating quality. After data sets 11 and 22 were recorded, the voltage curve of the Cu electrode significantly decreased, suggesting that the Cu had oxidized. Evidence of oxidation also occurred on the Cu coatings of the Cu-plated Al and the Cu-plated Fe electrodes. Moreover, destruction of the Cu coatings on the Cu-plated Al and the Cu-plated Fe electrodes altered the material exposed at the electrode surface. Therefore, the voltage curves of the Cu-plated Al electrodes and the Cu-plated Fe electrodes displayed more significant decreases, especially the Cu-plated Al electrode that had been artificially damaged prior to the experiment. Unsurprisingly, the voltage increased when using the Al and Fe electrodes with the Cu plating. However, the 10-μm-thick layer of Cu plating could not maintain the performance of the electrode for an extended period. Based on these results, one option would be to increase the thickness of the plating treatment, which is demonstrated in the supplementary experiment.

## Conclusions

The metal reactivity of an electrode has no significant effect on the voltage between a standing tree and its surrounding soil. However, passivation of the electrode materials markedly reduces the voltage. The conductivity of the metal electrode has an effect on the contact resistance of the electrode-soil and electrode-trunk contact surfaces, which significantly influences the voltage level. The metal with the higher conductivity leads to a smaller contact resistance, as well as to a higher voltage level. Therefore, the effects of metal conductivity on voltage are primarily determined by the surface metal materials in direct contact with the soil and the trunk. It is suggested that cheap metals, such as Fe and Al, can be selected for inclusion within a metal coating (with better conductivity), which would most likely achieve good efficiency, stability, and economy. Moreover, it is demonstrated that cheap nonmetallic materials, such as plastic, glass, wood, and ceramic, are also good choices for the inclusion. In addition, the thickness of the coating must be increased to obtain higher stability and reliability.
